# Strengthening implementation of diet-related non-communicable disease prevention strategies in Fiji: a qualitative policy landscape analysis

**DOI:** 10.1186/s12992-022-00859-9

**Published:** 2022-09-01

**Authors:** Sarah Mounsey, Gade Waqa, Briar McKenzie, Erica Reeve, Jacqui Webster, Colin Bell, Anne Marie Thow

**Affiliations:** 1 Menzies Centre for Health Policy and Economics, Faculty of Medicine and Health, Charles Perkins Centre, The University of Sydney, City Road, Sydney, NSW 2006 Australia; 2 Fiji National University, Suva, Fiji; 3 The George Institute, City Road, Newtown, NSW Australia; 4 Deakin University, Geelong, VIC Australia; 5 School of Medicine and Global Obesity Centre, Geelong Waurn Ponds Campus, Deakin University, Melbourne, Australia

**Keywords:** Diet-related non-communicable disease, Fiscal policy, SSB taxes, Policy analysis, Corporate political activity

## Abstract

**Background:**

Noncommunicable diseases (NCDs) are the leading cause of death globally, and the World Health Organization (WHO) has recommended a comprehensive policy package for their prevention and control. However, implementing robust, best-practice policies remains a global challenge. In Fiji, despite political commitment to reducing the health and economic costs of NCDs, prevalence of diabetes and cardiovascular disease remain the highest in the region. The objective of this study was to describe the political and policy context for preventing diet-related NCDs in Fiji and policy alignment with WHO recommendations and global targets. We used a case study methodology and conducted (1) semi-structured key informant interviews with stakeholders relevant to diet-related NCD policy in Fiji (*n* = 18), (2) documentary policy analysis using policy theoretical frameworks (*n* = 11), (3) documentary stakeholder analysis (*n* = 7), and (4) corporate political activity analysis of Fiji’s food and beverage industry (*n* = 12). Data were sourced through publicly available documents on government websites, internet searches and via in-country colleagues and analysed thematically.

**Results:**

Opportunities to strengthen and scale-up NCD policies in Fiji in line with WHO recommendations included (1) strengthening multisectoral policy engagement, (2) ensuring a nutrition- and health-in-all policy approach, (3) using a whole-of-society approach to tighten political action across sectors, and (4) identifying and countering food industry influence.

**Conclusion:**

Diet-related NCD policy in Fiji will be strengthened with clearly defined partner roles, responsibilities and accountability mechanisms, clear budget allocation and strong institutional governance structures that can support and counter industry influence. Such initiatives will be needed to reduce the NCD burden in Fiji.

**Supplementary Information:**

The online version contains supplementary material available at 10.1186/s12992-022-00859-9.

## Introduction

Noncommunicable diseases (NCDs) are the leading cause of death globally, representing one of the major global health challenges. The causes of NCDs are multifactorial, including upstream drivers such as globalization as well as more proximal factors [1]. To stem the rising NCD burden, the Member States of the World Health Organization (WHO) have committed to a comprehensive package of ‘best-buy’ policy recommendations [2, 3]. Investment into promoting healthy diets for reduced NCD burden provides the biggest return [4]. Interventions such as reducing salt intake through reformulation, front-of-pack labelling, education and awareness campaigns and banning trans-fats in the food supply chain figure highly. Also, the use of fiscal policy on unhealthy products – particularly sugar sweetened beverages (SSBs) - is a proven, cost-effective policy tool to reduce consumption of the taxed good [5–7]. Modelling studies have also indicated positive health gains from salt taxation [8]. However, achieving strong policy design and implementation for NCD prevention remains challenging, globally. In this paper, we examine opportunities to strengthen NCD prevention policy in detail in a single country, and reflect on learnings to improve action in this important global health policy area.

The Pacific Islands face a considerable NCD burden. The 2019 Global Burden of Disease (GBD) report indicated that Fiji, one of the largest countries in the region with a population of 911,000, had 84% total mortality and a 31% probability of premature mortality (defined as < 70 years) due to NCDs [1]. More specifically, the report indicated type 2 diabetes and ischaemic heart disease contributed to 23 and 21% of total, all-age mortality - the highest and second highest, respectively - in the WHO Western Pacific Region (WPRO). The most prevalent risk factors contributing to these mortality levels were high fasting plasma glucose (37%) and high blood pressure (25%) [1].

In the last decade, Fiji, as a WHO member state, committed to establishing and strengthening multisectoral national policies and plans for the prevention of diet-related NCDs. Additionally, in 2009/2010, two evidence-based governmental participatory research projects identified 22 policy actions for achieving healthier food environments and practices in Fiji and Tonga [9–11]. Recent, contextual evidence has demonstrated positive achievements for the implemented nutrition policies and actions that also align with the *WHO Global Action Plan for the Prevention and Control of NCDs 2013–2020* [2, 3, 12]. For instance, Fiji implemented taxation of unhealthy commodities to reduce consumption. In 2018, Fiji increased the import excise duty for SSBs to $FJ2.00/l (approximately $US 0.91) with a separate tax for locally produced SSBs of $FJ0.35/l (around $US0.17). Also, voluntary salt targets for a range of food categories have been agreed upon by the food industry with a view to reducing salt intake [13]. One specific multi-faceted intervention study combining behaviour change and industry reformulation between 2012 and 2016 resulted in a reduction of 1.4 g/day salt intake (weighted mean salt intake at baseline was 11.7 g/day and 10.3 g/day after 20 months) and while this was not statistically significant, subgroup analysis disaggregated by gender showed a statistically significant reduction in salt consumption seen by females from the Central Division (of 3.34 g/day; *p* = 0.017) [14].

However, the implemented policy changes in Fiji have been below the WHO recommendations (for an SSB tax, this is defined as a tax increasing consumer prices by between 20 and 50%; for salt, voluntary targets are for a 30% reduction in the mean population intake of salt/sodium) [15]. The impacts - both incremental and longer term - are yet to achieve the desired level of change; sugar and salt intake remain above WHO recommendations and SSB consumption is the highest in the Pacific Region [14, 16]. Thus, understanding and overcoming barriers to effective policy implementation has become a priority in Fiji.

Previous studies in the Pacific have demonstrated challenges to policy implementation including policy tensions, lack of engagement across sectors, minimal participation from industry and policy incoherence [17, 18]. Also, access to and use of evidence, consultation and stakeholder engagement, political dynamics, understanding trade policies, competing government priorities and problem awareness have been implicated [19–22]. An analysis of corporate political activity by Fiji’s food industry found they used numerous strategies and tactics to influence decisions and design of diet-related policy [23].

Our aim was to describe the political and policy context for preventing diet-related NCDs in Fiji and policy alignment with WHO recommendations and global targets (Additional file 1). To achieve this aim, the study objectives were to establish a comprehensive understanding of the current policy content and political context relevant to the prevention and control of diet-related NCDs, to analyse stakeholder interests and influence and to describe the food and beverage industry’s corporate political activity.

## Methodology

### Study design

We used a case study design that included:

1. Policy analysis: combining policy documentary analysis according to WHO policy recommendations for diet-related NCD prevention and stakeholder interviews
2. Stakeholder analysis: combining documentary analysis and stakeholder interviews
3. Industry corporate political activity: combining documentary analysis and stakeholder interviews

Case study research methods are recommended for analysing a naturally occurring phenomena over which the researcher has little or no control (so, in this research, diet-related NCD policy) [24, 25].

### Theoretical frameworks

Our study drew on four theoretical frameworks. Each were chosen to guide and inform our data collection and analysis in line with our study aim and objectives. The policy analysis and stakeholder interviews were underpinned by two widely used theories of policy making. The first was Shiffman’s theory, which focuses on political priority and identifies important influences as: the power of actors involved, ideas that frame an issue, political contexts and characteristics relating to a specific issue [26]. The second, Kingdon’s “multiple streams” theory, argues that for an issue to gain political priority on a government’s agenda, the problem, a set of policy solutions to the problem and political events are what lead to a ‘policy window’ that affects change [27]. Using this theory retrospectively can explain how particular health issues may (or may not) have gained political priority. The stakeholder analysis was developed and categorised using Varvasovszky and Brugha’s theoretical approach who define stakeholders as ‘*actors who have an interest in the issue under consideration, who are affected by the issue or – because of their position – have or could have an influence on the decision-making and implementation process’* [28]. The corporate political activity of Fiji’s food and beverage industry utilised the framework devised by Mialon and colleagues, which speaks to Shiffman’s theory in relation to actor power. Mialon’s framework focusses on the strategic actions used by corporations to influence public opinions and policy-makers [29], adapting earlier theoretical frameworks based on policy influence more broadly [30] and the tobacco industry [31] to be more targeted for food industry tactics [23].

Collectively, the different dimensions and key elements from each framework enabled us to understand the various barriers of policy implementation for diet-related NCD prevention in Fiji, and thus were useful to identify and inform opportunities to strengthen and scale-up WHO recommendations for policy action more completely.

### Data collection

We had two data sources for our study: (1) interviews with stakeholders, and (2) publicly available documents. The researchers involved in data collection and analysis have long, well established relationships with key policy makers in Fiji, particularly for salt reduction, NCD interventions and nutrition, as well as comprehensive experience in international nutrition, fiscal policy and NCD policy analysis.

We conducted interviews (*n* = 18) with key stakeholders and policy makers from within the relevant government sectors (health, finance, agriculture, education, employment), from industry, civil society, development partners and external actors who could contribute to a policy decision via cross-sectoral ‘policy networks’ [32]. Participants were identified from within each sector based on their role and level of involvement in policy making or implementation and engagement with the diet-related NCD policy process. Participants were recruited through in-country colleagues via email, in-person visits and telephone calls. The semi-structured interview guide was based on our policy theoretical frameworks, and asked about actor influence and power, institutional structures and governance, framing and beliefs (Shiffman) and also about perceptions and beliefs regarding diet-related NCDs as a policy ‘problem’ and policy ‘solutions’ (Kingdon), including recommendations in WHO Global Action Plans 2013–2020 (Additional file 1). Each lasted approximately 90 minutes, and were conducted by SM, BM, and GW. Interviews were conducted virtually, recorded and transcribed in full using NVivo transcription software. Validation of the data involved providing all five coders with a copy of the codes and codebook and discussing any discrepancies. See Appendix 1 for the interview guide. The coders SM, BM, ER, GW and AMT have experience in NVivo interview coding from projects involving comparable subject matter. The first two interviews were coded by all five coders and any discrepancies were discussed and resolved. The remaining transcripts were then coded by the first author (SM).

Publicly available documents were sourced through comprehensive internet searches of government websites, google and direct requests to relevant in-country actors until data saturation was reached. For publicly available policy material search terms were key words from WHO-recommended policy actions, including “multisectoral policy”, “fiscal policy”, “healthy food production”, “reformulation”, “food labelling”, “school food”, “food marketing”, “trade policy” and “tax”, and relevant sectors, including “health”, “trade”, “agriculture”, “gender”, “education” and “fiscal” [3]. Stakeholders were identified from policy documents, government websites and internet searches using the phrase: “NCD prevention in Fiji”, with the terms “NGOs”, “faith-based organizations”, “public-private partnerships”, “civil society”, “donor partners”, “academia” and “international organizations” (“EU”, “WHO”, “FAO”, “World Bank”, “JICA”, “AUSAID”, “SPC”), as well as common NCDs, such as “diabetes” and “CVD”. Search strategies for publicly available material for the corporate political activity component included internet searches for: “SSB and salty snack manufacturing and production in Fiji”, “food and beverage manufacturing in Fiji”, “local, transnational and multinational food and beverage corporations operating in Fiji”, and “importing and exporting food and beverage companies in Fiji”. Inclusion criteria for these components included: (1) internet material after 2015 (where feasible) and (2) documents in English.

We extracted and compiled the relevant content from the publicly available data sources for the documentary analysis component and for each study objective using an interim output in the form of Excel matrices, each underpinned by the relevant theoretical framework, similar to the approach used by Thow and colleagues [33]. The first matrix related to the policy content: key policy documents across all government sectors relevant to food and nutrition in Fiji (*n* = 11) were reviewed to identify current priorities and activities relevant to diet-related NCD prevention in this context. We extracted the policy content (Matrix 1) in line with key elements of Shiffman’s and Kingdon’s theories as well as drawing on the relevant WHO recommendations for diet-related NCD prevention (Additional file 1). The second matrix was developed for the stakeholder data which underpinned the analysis for determining stakeholder interests and influence in line with Varvasovszky and Brugha’s framework. The final matrix incorporated food and beverage industry presence and corporate political activity in Fiji, categorised according to the key elements of Mialon’s framework.

Validation of the final matrices was done collectively by the in-country co-author (GW), an independent leading in-country actor and the co-authors, discussing any discrepancies. Minor changes were made following the validation process.

### Data analysis

The data analysis was done in three stages: first, we analysed the interview data thematically; second, we analysed the documentary data in each of the matrices; and third, we conducted an integrated analysis across the different (analysed) data. First, we drew on Shiffman’s and Kingdon’s theoretical frameworks to derive and analyse thematic codes from the stakeholder interviews and the policy content from the matrix. Table 5 in Appendix 2 summarises and describes the NVivo-derived codes.

There was also an element of iterative analysis; the documentary policy data informed the interviews by providing specifics on the nature of the case study as the basis for questions on the agenda setting and policy processes. We used the two framework’s key elements to determine our findings: specific characteristics of the NCD issue; the most common framing and beliefs within policies and from interview participants’ perceptions relating to: (1) political priority, commitment and actions; (2) real and perceived actor interest, influence and power; and (3) the perceived problems and the solutions. We were then able to determine the nature of key underlying barriers and drivers for change in relation to diet-related fiscal policy, NCD policy more broadly and specifically, actions relating to WHO recommendations for diet-related NCD prevention.

The stakeholder analysis data in the matrix were used to map out levels of interest of each stakeholder, relevant to prevention of diet-related NCDs, their position relevant to this issue, and their level of influence, following the approach described by Varvasovzsky and Brugha [28].

The corporate political activity analysis used the data in the matrix to identify mechanisms through which industry sought to influence the policy process. These were described and categorised with reference to Mialon’s framework [23].

We then triangulated the emerging themes from the stakeholder interviews with our findings from documentary policy, stakeholder and corporate political activity analyses [25]. The integrated, manifest content analyses were discussed among the authorship team, and refined. The major themes emerging from the integrated analysis were the characteristics of the multisectoral policy landscape, and opportunities to strengthen policy processes to enable effective policy, stakeholder dynamics and the influence of industry, and corporate political activity and behind-the-scenes influence.

## Results

We found the policy landscape in Fiji to be comprehensive and multisectoral (Table 1). There are robust NCD prevention plans within the Ministry of Health’s policy framework however, challenges remain, particularly in relation to the limited attention to multisectoralism in sector plans and the current lack of institutional structure. Although there are a vast number of policy stakeholders involved in the policy process, we noted limited engagement from civil society and the general public. Also, key stakeholder dynamics have been characterised by a dominant influence from industry whose corporate political activity to influence policy has primarily been undertaken by the 12 main food and beverage industry actors in Fiji. There is alignment with WHO recommendations for policy on NCD prevention however these could be strengthened to support the achievement of global targets (Table 2).

**Table 1:**
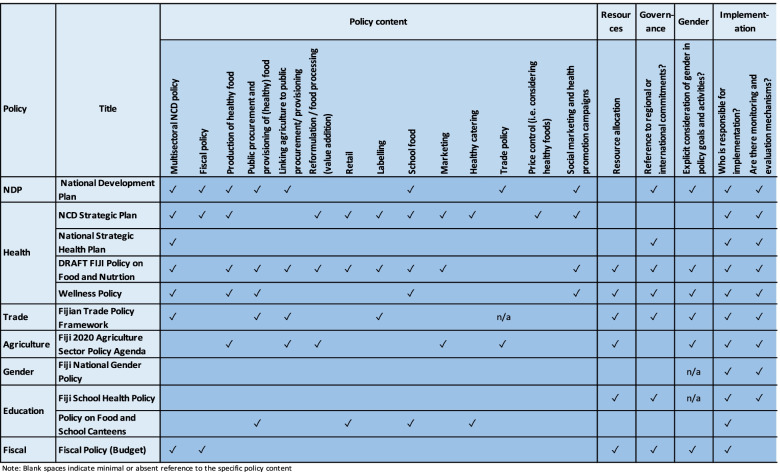
Policy content for NCD-prevention against WHO Global Action Plan recommendations

**Table 2:** Documentary policy analysis findings relevant to factors WHO considers in recommendations for NCD prevention

	Aspect of policy content^a^	Policy analysis finding
**Policy content**	**Multisector policy approach**	• All analysed policies acknowledged NCD burden in Fiji. Six policies specifically mentioned multisectoral action was needed for reduced NCD burden, including the overarching National Development Plan (NDP), four from the Ministry of Health and Medical Services (one is in draft form and yet to be endorsed).
	**Fiscal Policy for NCD prevention**	• The NCD Strategic Plan and the Fiscal Budget reports clearly mention fiscal policy as an NCD prevention strategy. The Government of Fiji have in place a range of targeted taxes to reduce the consumption of less healthy products high in sugar, salt and fat.
	**Production of healthy food**	• The NDP indicates that the production of healthy, local food is a priority for Fiji’s food and nutrition security. • The NCD Strategic Plan mentions ‘backyard’ gardens, and the draft Policy on Food and Nutrition Security provides specific activities and targets to enhance and promote healthy, sustainable, diversified and resilient food systems. The Wellness Policy indicates the influence of the Ministry of Education could enhance gardening activities in the school setting. No other policies mention the production of healthy foods.
	**Reformulation/food processing**	• The NCD Strategic Plan and the draft Policy on Food and Nutrition Security mention reformulation efforts to reduce salt, sugar and fat in locally manufactured products.
	**School food and guidelines**	• The NDP includes a priority to increase interaction and involvement of schools to encourage the younger generation to be more food secure. A current programme includes 84 schools undergoing health promotion awareness. Beyond these strong initiatives for school programmes, four other policies mention school food and canteen guidelines, coming from Ministry of Education and Ministry of Health. The policies from Education have been confirmed to be current, however, there are no timelines or indicators, no review date and no mention of budget/resources or accountability/responsibility.
	**Marketing**	• Two of the 11 policies mentioned marketing regulations (NCD Strategic Plan and the draft Policy on Food and Nutrition Security). Both relate to the adoption and implementation of marketing of non-alcoholic beverages and foods to children as well as enforcing the existing regulations regarding misleading advertising.
	**Trade**	• The Trade Policy briefly mentions diet-related NCDs: ‘*In addition to global concerns over health issues surrounding tobacco products, Fiji and other Pacific Islands have concerns over trade in products that are seen as potentially increasing susceptibility to NCDs.’*
**Framing and beliefs**	**Nutrition Promotion**	• Four of the 11 policies mention health promotion. The overall National Development Plan indicates the importance of food and nutrition security, including measures such as local media campaigns, corporate and civil society to engage in initiatives to encourage consumption of local produce with the promotion of recipes etc., particularly in primary, secondary and tertiary educational settings. The NCD Action Plan has the additional focus of encouraging schools to be safe places for active play. • Both the draft Policy on Food and Nutrition Security and the Wellness Policy contain comprehensive strategic actions to enhance and increase knowledge and the promotion of healthy diets and lifestyles to reduce NCDS throughout the lifespan. • All 11 policies analysed alluded to NCDs being a health issue in Fiji. Reasons included NCDs being the leading cause of mortality, morbidity and premature morbidity, the impact on the economy, labour supply and the impact from healthcare costs on government and household budgets. • From our analysis two sectors (Health and Economy (NDP)) explicitly described causes of NCDs. • Identified risk factors were biochemical (e.g., blood glucose, lipids), high BMI, an aging population and changing dietary patterns. The draft Policy on Food and Nutrition Security made it clear the food system was the primary driver that led to poor individual consumption choices as well as the high price of local food as a result of reduced food security. Furthermore, the Wellness Policy stated that beyond these underlying drivers, an issue in Fiji is that although wellness is widely promoted for reduced NCDs, it is not understood. • Five of the six policies outlined those responsible for NCD actions and indicated a multisectoral approach. The sixth (Education) indicated that policy implementation was the responsibility of the school head and canteen operators. The Wellness Policy added the community and individuals also needed to take action and the National Health Strategic Plan indicated there should be health-in-all-policies. The NDP briefly indicated a multisectoral approach was required. • No policy discussed the role of the state in regulating markets. • Seven policies gave indication of the most effective policy response for NCDs. The NDP (Economy) gave a general response to suggest ‘*reduced premature mortality rates in line with international targets*.’ The Ministry of Health’s three policies indicated various responses. For instance, the NCD Strategic Action Plan gave a high-level response of ‘*following WHO’s menu of options within the Global NCD Action Plan to achieve the nine targets*,’ whereas the National Strategic Health Plan championed the more local ‘*Healthy Islands Framework*’ as being significant in influencing the current approach to NCDs. This approach utilises a settings approach as well as a ‘*Wellness*’ concept. The Wellness Policy also adopts this ‘*Wellness*’ concept but goes an extra step to suggest holistic wellness that incorporates social, spiritual, environmental, occupational, psychological, financial and physical wellbeing. Two policies mentioned multisectoral engagement is essential.
**Resources**		• Our analysis provided mixed results. Seven policies mentioned budget and resource allocation and of these, only two polices (draft Food and Nutrition Security Policy and the Fiscal Budget Address) gave defined amounts and sources. Of the remaining five, high-level statements indicating the budget will come from the ‘public health budget’ (Education), “partner ministries’ (Health), ‘requests are being made to international donor partners’ (Agriculture) or that the ‘government will allocate resources’ (Trade). Four policies provided no mention of budget or resource allocation. More importantly, the National NCD Strategy and the NDP gave no mention of budgetary allocation (the NCD policy had an empty ‘budget’ column in their indicator table).
**Governance**		• Our analysis found all policies suggested NCDs and their prevention strategies should fall under the Ministry of Health and Medical Services. The exception is within the draft Food and Nutrition Security Policy which had a multi-stakeholder, multisectoral, high-level committee to provide a mechanism for reporting implementation activities and outcomes (however, as this is not yet endorsed, there is currently no high-level committee). Because Trade, Agriculture, Economy and Gender do not address NCDs in their policies, there was no mention of coordination or responses or international commitments to reducing NCD burden and each sector indicated responsibility for their own policy.
**Gender & equality**		• We found within nutrition-relevant policies, there was an emphasis on the need for equal opportunities in education, social services as well as health. While there was some inclusion across sectors for improving opportunities for women (e.g., In the Trade policy and Agriculture policy), this related largely to improving the economic position of women in Fiji.
**Implementation**		• The sector specific policies had clear allocation of responsibility to the relevant line ministry, and all line ministries report to the National Development Plan. Two Health and Medical Services policies indicated a multisectoral accountability (draft Food and Nutrition Security Policy and Wellness Policy). Both provided details to suggest accountability was given to the leading sector given responsibility for the action or initiative (e.g., Agriculture for food security targets, Education for school canteen targets, etc.). However, the mechanism for ensuring implementation could have been clearer. • Nine of the 11 policies included in our analysis provided M&E mechanisms. For instance, the M&E indicators relating to NCDs across the Ministry of Health’s policies were mostly robust and have given clear targets and timelines for evaluative analysis. This included the National Health Strategic Plan, the NCD Strategic Action Plan, the Wellness Policy and the draft Food and Nutrition Security Policy. The latter had a complex multisectoral system for implementation and accountability across sectors and clearly defined each. • Similarly, the Agricultural Sector Policy Agenda described four stages of the implementation, where evaluation reviews were to be carried out mid-term and mid-2020 for purposes of ‘improving the existing policies to ensure continuity and sustainability.’ No details of how these would be carried out or by whom was included in the policy.

^a^Source: Based on authors’ analysis. Policies identified by authors were: Ministry of Economy: National Development Plan (NDP), Fiscal Policy Budget address; Ministry of Health and Medical Services: National Health Strategic Plan, NCD Strategic Plan, Draft Fiji Policy on Food and Nutrition Security, Wellness Policy; Ministry of Trade: Trade Policy Framework; Ministry of Agriculture and Fisheries: Agriculture Sector Policy Agenda; Ministry of Women and Youth: National Gender Policy; Ministry of Education: School Health Policy, Policy on Food and School Canteens

### Multisectoral policy strengths and opportunities

Fiji has a 20-year (2017 to 2037) National Development Plan (NDP) which is translated into individual sector development plans with comprehensive and inclusive activities to be implemented in five-year blocks. At the heart of the NDP is ‘*socioeconomic development for every Fijian*,’ aligning with global targets in the Sustainable Development Goals (SDGs) and the Paris Agreement on Climate Change. As of November 2019 there were 11 active policies relevant to diet-related NCD prevention from Economy, Health, Agriculture, Trade, Education and Women and Youth (Table 6 in Appendix 3).

Comparing these policies with the frameworks of Shiffman and Kingdon, we identified three key opportunities for strengthened diet-related NCD prevention relevant to the policy process. These were: (1) the potential for increased multisectoral engagement, governance and institutional structures (2) to include more transparency around budgetary and resource commitments and allocation, and (3) greater commitment to enforcement, monitoring and evaluation mechanisms of current regulatory and fiscal policies (e.g., excise taxes on SSBs).

#### A multisectoral approach is needed

Multisectoral action was mentioned in almost half of the analysed policies and highlighted by policymakers, who also reported that engagement of non-health sectors was limited. For example:

*“.... Having a whole-of-government approach or a whole-of-government policy that cuts across all sectors, that will include the private sector. Major players in the private sector would also include the food industries. It will include civil society organizations, the NGOs and academic institutions, as well. So, everyone has to pitch in to this policy....”* Civil Society.

However, some participants explained that non-health sectors often fail to engage in implementing policies related to NCD prevention:

*“.... I’m not sure what the others would say, but definitely from my perspective, there’s been limited progress around multisectoral collaboration. The different sectors are still kind of working in isolation****....”*** Development Partner

Poor governance was a barrier to multisectoral action. There was no multisectoral governance or coordinating institutional mechanism overseeing implementation of the national NCD strategy in Fiji:

*“.... Well, there should be a formal kind of structure to be able to monitor what is going on in terms of the food and nutrition security. Like, something central so that they are able to say, OK, these people are doing this, this group is doing that, so there is no repetition, and you can allocate resources efficiently...”* Government

#### Transparency in resourcing (commitments and where the money should come from)

Inadequate budgetary and resource allocation was a challenge. Seven policies mentioned budget and resource allocation and of these, only two polices (Draft National Fiji Policy for Food and Nutrition Security and the Fiscal Budget Address) identified defined amounts and sources. Of the remaining five, high-level statements indicating the budget will come from the ‘public health budget’ (Education), ‘partner ministries’ (Health), ‘requests are being made to international donor partners’ (Agriculture) or that the ‘government will allocate resources’ (Trade). Four policies provided no mention of budget or resource allocation and critically, the National NCD Strategy and the NDP did not include budget allocations (the NCD policy had an empty ‘budget’ column in their indicator table). Interview participants, from both inside and outside government, pointed to the lack of sufficient budget allocation as an example of a lack of genuine commitment. For example:

*“I think it’s not a priority [NCDs and nutrition]. If you look at the budget, even within the Ministry of Health, you can see that the budget is very low for nutrition....”* Development Partner

#### Monitoring and evaluation of active policies

Shiffman’s priority setting framework highlights the need for monitoring and evaluation to help enforce policy. Participants identified the absence of monitoring and evaluation contributing to a lack of evidence regarding severity of the problem and the (potential) effectiveness of specific policies. However, there were participants who indicated substantial efforts were being made (Table 4). 

*“.... I think it’s the monitoring that’s also a big issue in Fiji. We have the policies in place, but actually monitoring them. There definitely needs to be policy around this...”* Development Partner

### Stakeholder dynamics and the influence of industry

We identified seven groups of stakeholders with an interest in NCD prevention in Fiji. These were (1) government, (2) industry, (3) academia, (4) civil society, (5) development partners, (6) non-government organisations (NGOs), ‘other’ (e.g., faith-based organizations, community), and (7) media. Stakeholder groups had varying levels of interest in the NCD issue which ranged from ‘low’ interest to ‘high’ levels of interest (Table 3). Industry was the only group consistently perceived to have low interest in the NCD issue. Similarly, there was variation across the respective groups as to the level of support they provided for NCD action, ranging from ‘completely supportive’ to ‘competing priorities’.

**Table 3:** Summary of documentary stakeholder analysis^a^

Stakeholder	Main activity or focus area	Perceived level of interest in NCD prevention	Position (supportive, neutral, competing priorities	Perceived level of policy influence
Development Partners	To provide technical expertise and assistance, capacity building, financial and human resources for a variety of national issues, including health	Medium-High	Supportive with competing priorities	High
Government	Responsible for overall strategic direction of the country across sectors	Low-Medium	Supportive with competing priorities	High
Industry	Manufacture and supply, both domestically and internationally, of food and beverage products	Low	Competing priorities	High
NGO’s, faith-based organizations, community	Provide advocacy, innovative health and education initiatives and community/household support for NCD prevention	Medium-High	Supportive with some competing priorities	Low-High
Civil Society	Provide community services, advocacy, capacity building and health promotion for reduced NCD burden	Medium-High	Supportive	Low-High
Academia	National and international institutions provide dedicated research and evidence for informing policy.	Low-High	Neutral-supportive	Low-High
Media	Media coverage of NCD prevalence, prevention strategies and health promotion events connected with the issue	Low-Medium	Supportive with competing priorities	Low-High

^a^Categories derived from Varvasovsky and Brugha’s theoretical framework. Each ranking for interest, position and level of policy influence is the authors’ analysis, validated by in-country colleagues and does not necessarily signify opposing or negative positions

Source of stakeholder data: Internet/website/online media content

Overall, policy documents attributed influence and power to government and development partners because of their extensive provision of funding and technical advice. However, from our 18 stakeholder interviews, no government participants alluded to the power of development partners and only one respondent spoke of development partners being influential or wielding significant power on decision-making:

*“.... Sometimes the development banks like the World Bank or the Asian Development Bank. They come with big packages and a commitment for the country, because the country has to repay, and they have their own way of doing things and packaging things and I think they have an influence on how the development agenda prioritizes things....”* Development Partner

Participants from government ministries and civil society perceived the food and beverage industry to have the most influence on the policy process and decision-making – as illustrated by the following quote:

*“.... Right now, the food industry is the biggest influence. And the big ones, the big names. They come to us [Government] and they understand the situation but change it into their stories. I mean, they are for business...”* Government

To achieve a more balanced distribution of power, several interview participants indicated a whole-of-society approach would be most beneficial. Participants perceived government, in the quest of continued support, would be more likely to successfully implement policy actions when communities, civil society, and NGOs are involved in consultations. When asked who should be involved in NCD policy processes, one respondent replied:

*“.... I think the key enabler is the community or those stakeholders more at the operational level, rather than at the government level. It is not that government is against it, it is just that it’s lower down in their priority list and I don’t think we should wait for them to pick it up. We can push it up from the operational level or from the stakeholders rather than waiting for big government decisions before we implement....”* Civil Society

### Corporate political activity and behind-the-scenes influence

We identified 12 businesses/companies manufacturing or selling food and beverages in Fiji. Five were beverage industries, operating under one large corporation (Fiji Beverage Group) and included the transnational corporation of Coca-Cola. Seven were food industries and included two transnational corporations, Nestlé, and the Carpenter Group. Nine were 100% Fiji owned. Of these, four were state interests (Copra Millers, Food Processors, Fiji Sugar Corporation and Fiji Meat Board), and one was a large, private corporation (Punja’s, or FMF).

These Companies positioned themselves on their websites and in the media as making a substantial contribution to the economy of Fiji. Based on data from company and accountancy websites, we estimated that the food and beverage industries collectively employed over 11,600 people in 2018–2019. If the total number of people employed by the Fiji Sugar Corporation are included in this estimate, it increases to a total of 211,600 people or nearly a quarter of Fiji’s population. Fiji Sugar Corporation alone, directly employed 2000, Coca-Cola employed 1100 people (including Paradise Beverages) and Nestlé employed approximately 200 people.

We estimated the Fiji Beverage Group (FBG) generated nearly US$1B for the Fijian economy, Goodman Fielder nearly US$115 M (2017), Punja’s approximately US$87 M (2019) (another source suggested a more modest amount of US$12 M). The two Nestlé factories generated over US$12 m in export sales annually. Furthermore, Nestlé has invested F$20 million in Fiji since it transferred manufacture of an instant noodle line from Nestlé New Zealand to a factory in Ba, north Viti Levu (2018).

Table 4 provides a summary of strategies developed by Mialon et al. [23]. From our corporate political activity analysis, and across the 12 industries identified, we found 11 used the ‘constituency building strategy, nine used the ‘information and messaging’ strategy, and one used the ‘policy substitution’ strategy.’ The FBG used all three strategies, and five industries used two strategies. From our desk-based analysis, we found no examples of the ‘financial incentive,’ legal action’ or the ‘opposition fragmentation and destabilisation’ strategies occurring.

**Table 4:** Key corporate political activities

• Information and messaging: e.g., lobbying, reframing the debate, stress economic importance of the industry • Financial incentives: e.g., funding political parties • Constituency building: e.g., Establishing relationships with key leaders, policy-makers and organizations and media, seek involvement in the community • Legal strategies: Use (or threat) legal action against policies or opponents, influence development of trade and investment agreements • Policy substitution: voluntarily reformulate some foods (least consumed), promote healthy diets • Opposition fragmentation and destabilisation: creating disconcerting thoughts by those opposing the issue Industry may utilise one, some or all of these strategies to thwart policy action.	

Source: Mialon [23]

#### Constituency building

Constituency building was the most widely used strategy (defined in Table 4). Evidence indicated that eleven of the 12 corporations had implemented various initiatives to establish community involvement and support, and to establish relationships with influential actors. The most frequently applied constituency building strategy was marketing, through sponsorship of sports events, buildings, and equipment (four corporations) and cultural activities within the community (three corporations). To illustrate:

*“One example is the Coke games, which involves the secondary schools. So, what happened during these games? There’s only distribution of Coke during that game. So instead of water, only Coke or products from Coca Cola is being distributed....”* Civil Society

The beverage industry was involved in several initiatives beyond these including supporting gender-related initiatives, environmental recycling programmes, food baskets for the food-insecure and those below the poverty line, and natural disaster relief programmes.

#### Information and messaging

Information and messaging were the second most common strategy (defined in Table 4). Most messages from both the documentary analysis and stakeholder interviews framed dietary choices as individual choices or responsibility and highlighted the contribution of their products to a ‘wide and varied diet.’

*“Sugary drinks are one of the culprits which people love to consume here in this country and around the world. But drinking that in moderation, I believe it would help quite a lot. And I believe that is the case with the food. Also, because there is something like portion control, which Fiji doesn't follow.....”* Food Industry

There was evidence of ‘framing’ in some responses to our interviews. For example, one participant deflected attention from their own company by pointing to other products and/or external factors as the ‘culprits’ for NCD burden. Examples were supermarket ‘specials’, the need for education and other lifestyle factors:

*“.... Alcoholic beverages are not very good as regards to the NCD concern. People eat a lot of red meat with it and the quantum of red meat which they eat during a drinking session is very substantial. So, alcohol I would say is causing much of the damage, but the red meat consumed with it is causing all that and more damage....”* Food Industry

Company websites also indicated the importance of ‘physical activity’ as part of a holistic approach to a healthy lifestyle “*for a healthier future and improved quality of life*”. Other company websites highlighted the beneficial nutrients in their products (e.g., Fortified flour, coconut oil) or organic, non-genetically modified and gluten free products (e.g., sugar). Some also provided colourful, appealing recipes using their products. One drinks manufacturer claimed they were encouraging responsible marketing to children across the sector and stressed the importance of their presence to the Fijian economy but without citing specific examples.

Two interview participants from industry advocated their adherence to food manufacturing standards and regulations. For example:

*“.... We follow all the guidelines from government. We also do the nutritional panel. If we export products to the US and Canada, we follow the FDA guidelines.....”* Food Industry

Furthermore, our industry stakeholder participants framed their involvement in the NCD issue - as well as actions taken by other stakeholders - in a proactive and positive manner:

*“We have been taking all initiatives [to improving the production process and to export quality products] and the government is quite appreciative of all the things which we do....”* Food Industry

#### Policy substitution

Only the beverage industry (Fiji Beverage Group) had information publicly available for specific actions to invest in healthier products through reformulation and policy substitution (defined in Table 4). However, the companies under the Fiji Beverage Group represented a large proportion of the food and beverage sector and these findings showed their tactics are the most comprehensive in scope.

One local food industry participant indicated their company would be willing to spend money on new products that benefit society if incentivised by the government:

*“.... I believe if research is done to find out, for example, if oatmeal really helps to improve their dietary pattern of people. Then, if Fiji doesn't grow oats as such, and they have to be either imported from Australia or Canada or from Europe what would happen?If the duty got to zero, then the oat products become more affordable, yes. Similarly, equipment to manufacture a particular cereal if zero duty is there and we've been told, OK, why don't you install this plant? Why don't you put money behind it? And promote this product, it will really help, really benefit the society. Yes, I believe a company like us would do it....”* Food Industry

#### Opposition fragmentation and destabilisation

We did not find any direct evidence of opposition fragmentation and destabilisation. However, it was perceived by most participants to be a major barrier to NCD policy implementation:

*I think industries [have most influence], especially the big industries. They play a strong role in their position. Like, we have experienced this before where there was policy that was going to come out. We would try to do consultations and this industry went around the back, talked with the Minister and that changed everything - it didn’t go through. You know, those are the sort of things that happen. We’ve had two cases like that. The not so big industries tend to follow the formal consultations. It’s the big industries and they have their own way of handling things....”* Development Partner

*“When we sit in meetings, oh, it’s a beautiful meeting. Until, after that meeting, what happens informally backstage or whatever. That’s when the politics of it comes in. Behind the scenes....” Government.*

Several stakeholders (from industry, civil society, government and development partners) indicated that they saw industry involvement in policy design as an ‘enabler’ for addressing NCDs. These participants suggested that although industry was part of the problem, they could also represent a link enabling a long-term solution:

*“.... I would suggest getting the industry to come because they would be the enabler....”* Civil Society

A stakeholder from the food industry confirmed their support for reducing NCDs in Fiji, but suggested resourcing towards the agents of change was a challenge, generally, within the Pacific region. This resourcing included both from the industry itself but also, from government funding:

“.... *I think that whenever we’re [industry] invited to be involved in discussions or workshops, then we are very supportive of it. But ongoing lack of resources can make it a bit difficult to organize, and resourcing appropriately can be really challenging with a lack of funding. It can be difficult to get off the ground compared to more established markets or countries....”* Food Industry.

## Discussion

We found a robust landscape of NCD-prevention policy in Fiji that could be strengthened with attention to the policy processes surrounding alignment with WHO recommendations, coordination, coherence, implementation, and evaluation. Our research confirms previous findings on the barriers and facilitators of policy innovation to address diet-related NCDs by the government of Fiji and extends the previous work by examining the body of policy for diet-related NCDs as a whole. For instance, descriptions of the nature of the policy, the political environment, industry influence and the need for a whole-of government approach described in Latu et al. (2018) resonated well with our findings [12]. We were able to show similar examples but for different policies in the current policy context. Also, Teng et al. (2021) acknowledged the current taxes on SSBs in Fiji [34]. We confirmed these findings and described potential barriers to further scale-up of diet-related NCD prevention policy more broadly, including the use of taxation. We identified a large number of policy-relevant stakeholders spanning seven stakeholder types, with varying levels of interest and priority for diet-related NCDs. Most influential were government and industry actors, the latter showing evidence of corporate political activity.

Our analysis identified several opportunities for strengthened policy action in Fiji. First, translating the clearly articulated multisectoral approach in the NDP into sector policies. Drawing together all current NCD-related policy in Fiji allowed us to identify that multisectoralism was missing or weaker in sector specific plans and that there is no current governance structure (e.g., a multisectoral NCD steering committee) to support coordination mechanisms for NCD prevention. Literature shows that policy actions are more likely to be implemented when guided by strong multisectoral governance and institutional structures which engage and hold accountable all actors of a policy process [26, 35].

The second key opportunity was to identify evidence-based, contextual strategies, such as monitoring and evaluation, for improved nutrition in the face of competing priorities. This opportunity has been identified through other studies. For instance, a new study from Bangladesh demonstrated how it is possible to address extreme weather events or climate shocks using epidemiological surveillance data (e.g., seasonality), while integrating nutrition policy and food system weak spots to anticipate nutritional requirements throughout the seasons [36]. Similarly, evidence from a policy analysis in India opened the way to integrate under- and over-nutrition policy priorities as well as increase the production of healthy produce within India’s food system [37]. Also, in response to high intakes of SSB consumption in the Solomon Islands, the use of specific evidence on the health and economic impacts of an SSB tax supported advocacy coalitions to exploit policy opportunities for SSB taxation packaged in a contextual, multisectoral policy design [38]. Against this backdrop, our findings suggest that providing evidence-based, contextual NCD-related policies in the form of multisectoral, nutrition-sensitive policies would not only target direct, cross-sector policy issues such as increased agricultural production and economic growth in Fiji but would also allow incremental public health improvements over time. Outcomes of such an approach could lead to a workforce and population that is more resilient to climate and health shocks while concurrently maintaining and following through on commitments for improved nutrition. Cross-sectorally, this translates to the fact that the National Development Plan’s target of increased commercial agriculture for the export market need not leave behind the ‘stated’ goal of reducing consumption of sugar, salt and fat and improving nutrition and food security. Increased trade liberalisation can include importation of healthful products (in Fiji, fruit and vegetable imports were removed by the government in a bid to prevent obesity and NCDs), and increased food and beverage manufacturing should include more reformulated, healthful dietary options with reduced sugar, salt, and fat [39].

More explicitly, for Fiji, the global and local changes due to the COVID-19 pandemic could align well with the upcoming endorsement of the National Fiji Policy for Food and Nutrition Security, in addition to the country's overall strategic direction of economic growth. This ‘policy window’ would allow government to consider the previous policy’s effectiveness, future challenges for nutrition and NCDs in light of competing priorities such as shocks of pandemics or extreme climate events which occur annually in the Pacific region. However, in the short term, population nutrition outcomes in the Pacific region remain particularly vulnerable to trade shocks and supply disruptions, such as those catalysed by the pandemic [40]. While trade liberalisation can reduce supply shortages and foster economies of scale, it can also increase access to unhealthy food imports. This is exemplified by the reversal of the trade ban on turkey tails upon Samoa’s accession into the World Trade Organisation in 2008, a high fat product banned for its contribution to the NCD crisis [41]. Increased market share of unhealthy products is likewise associated with a decline in the availability of traditional food items, further accelerating the NCD burden [42]. In sum, incorporating fresh, current, global and local evidence across sectors benefit not only the next set of nutrition policy actions to align with WHO recommendations and voluntary targets, but also ensures maximum impact for diet-related NCD prevention.

Stronger governance and institutional structures are critical. Such governance (e.g., a multisectoral NCD steering committee) ensures necessary inputs are embedded in the policy process for cross-sectoral policy coherence to become agents of change. Our findings align with evidence that indicate political commitment is more than an agenda-setting exercise; instead, others have argued adequate authority, adopting polices, allocating resources, and coordinating outcomes through efficient monitoring and evaluation is required for ‘*as long as necessary to get the job done*’ [26, 35, 43]. We identified that although political *will* for policy implementation is strong amongst policy-makers, what is required by advocates is framing the NCD problems in light of the wider government’s strategic direction for economic growth. This aligns with Shiffman’s theory on policy priorities and the ‘external frame’ in which ‘*those involved in the issue understand and portray it*,’ and specifically, ‘*the framing of the issue in ways that resonate with external audiences, especially the political leaders who control resources*’ [26]. With this approach, we see significant opportunities for closing the gaps and leveraging off the previous work done by the Ministry of Health to foster increased commitment for improved nutrition in Fiji.

Our findings relating to corporate political activity of the food and beverage industry in Fiji provide more recent examples of similar activities previously observed by Mialon, but were triangulated with in-depth industry stakeholder interviews [23]. The strategies employed by industry create tension in achieving NCD reduction, because it is assumed that reducing NCDs means consuming less of the products these companies produce yet achieving profits means selling more. Or more simply, government decision-making is in tension with the market-driven economy if government is imposing regulations and fiscal policy that impact consumption patterns. Similar policy landscape analysis in sub-Saharan Africa has also identified tensions between economic policy priorities and economic interests, and adoption and effective implementation of diet-related NCD policies [33].

How can these tensions be overcome? Available literature points to the lessons learned from (1) challenging ‘Big Tobacco,’ and the achievement of the Framework Convention on Tobacco Control (FCTC), and (2) strategies that helped achieve the International Code of Marketing of Breast-milk Substitutes [44]. Here, and similar to our findings, literature indicated the challenge of holding industry to account and the agency provided by a united policy community and particularly, the role of civil society (thus, the opportunity for a whole-of-society approach) [20, 26, 44]. However, civil society is likely to face challenges in holding (multinational) industry to account and it is important for governments to work together with civil society to strengthen accountability.

In contrast to this, there is an increasing trend to favour partnership approaches with industry actors for the health sector, particularly in countries with resource constraints [44–46]. Our findings show that the discourse of some interview participants from civil society supported this approach. However, while the opportunities of engaging in these partnerships in a bid towards achieving global health goals are appealing, the effectiveness of these partnerships to improve population health related to NCDs is widely debated [45]. Cautious evaluation and a sound understanding of potential conflicts of interest as well as having clear criteria for regulation and accountability prior to any agreed engagement must be implicit in order to maximise benefits and mitigate the risks [45, 46]. In terms of managing conflicts of interest, clear communication of policy goals and stakeholder roles, transparency mechanisms, balanced representation and strategies for mutual accountability and monitoring are essential. More information on identifying and preventing conflicts of interest see [47–50].

Our final observed opportunity is strengthening the influence of grass-roots actors. Influence can either facilitate or antagonise effective implementation of whole-of-government NCD-related policies, and our research demonstrated that there is sufficient willingness from most actors to take action on NCDs. Our findings have demonstrated that Fiji can facilitate effective policy change for diet-related NCDs by embracing and capitalising on a whole-of-society approach, where individuals, communities, civil society, academics, policy advocates, development partners and international organizations join forces to provide a unified voice and contribute to the policy process.

Our research forms part of an extensive, multi-pronged research grant being undertaken to support the strengthening and scale up of food policies in the Pacific region [51]. Collaborative engagement around the findings captured in this component of the research between government and non-government actors and researchers now provides the opportunity to explore more fully the drivers of the challenges identified but also ways to exploit identified opportunities that can benefit the policy process, and thus, improve health and economic outcomes for NCD burden.

### Strengths and limitations

A strength of this study was the triangulation and integration of documentary data and interviews with key actors within theoretical frameworks for contextual insight into the policy process in Fiji. Another strength is that we captured the views of actors directly involved in policy implementation in Fiji. Our research also had limitations. First, our documentary analysis relied on publicly available information that can be incomplete and may not reflect all current policy practices. Policy analysis is a sensitive area, and some documents may not be accessible. To overcome this limitation, we searched documentary sources until we reached data saturation and triangulated the data with stakeholder interviews and validation with our in-country colleagues. However, enquiry and observation are inherently shaped and filtered by the values and positionality of the researchers. The research team included one in-country researcher (GW), however it was important for the rest of the team to recognise their positionality as external to the Fiji country and context; failing to reflect upon this may have compromised the applicability of the research findings. Collaboration with in-country colleagues, consultations, and triangulation of research methods effectively minimised this threat of bias. A further limitation is that we had a purposive sample of actors and may have missed perspectives from key groups (e.g., within civil society). Another limitation was that the insights provided by interview participants were subjective and may not be comprehensive. This is particularly relevant for the corporate political activity findings where certain practices and strategies may not be publicly acknowledged. We sought to overcome this limitation by gaining additional insights from relevant stakeholders through confidential interviews.

### Further research

Our research adds to the literature by providing an important platform for further, more targeted analyses by public health researchers in partnership with relevant ministries or development partners. For example, new research could assess impacts of scaled-up taxation, and most useful would be the impacts on children and adolescents as evidence globally is limited. Also, the potential impacts of earmarking revenue generated for WHO recommendations and policy options for improved health awareness, and/or the health outcomes of sustained and effective policy actions targeting salt, sugar, and fat reduction strategies. Cost-effectiveness studies of fully implementing WHO policy options would benefit ministries and be a valuable addition to the literature. This comprehensive policy landscape analysis may provide a useful ‘template’ for other countries or similar settings in the face of conflicting policy priorities, particularly when integrated with stakeholder interviews and the analysis of industry activity.

## Conclusion

Opportunities exist to strengthen and scale-up NCD policies in Fiji. These include (1) strengthening multisectoral policy engagement, (2) ensuring a nutrition- and health-in-all policy approach is adopted to simultaneously address reducing the consumption of high sugar, salt and fat products to help reverse NCD burden as well as competing policy priorities, (3) using a whole-of-society approach to strengthen political action across sectors, and (4) identifying and countering food industry influence. Also, more clearly defined roles for government, responsibilities and accountability mechanisms, clear budget allocation and strong institutional governance structures that can manage industry influence.

### Supplementary Information

**Additional file 1.** WHO policy recommendations for NCD prevention. Source: WHO (2017) [3].

## Data Availability

The datasets used and/or analysed during the current study are available from the corresponding author on reasonable request.

## Appendix 1

### Interview Guide

**Overview of interview objectives:**

- Policy-relevant beliefs and frames and sectoral priorities
- Stakeholder power and mechanisms for influence
  - Industry mechanisms for influence – use Mialon framework for prompts

***This interview is designed to complement the documentary data collection for the policy landscape analysis in Fiji (policies, stakeholder analysis and industry corporate political activity analysis). The documentary data will inform the prompts etc used throughout –*** **e.g.** ***, specific policy sectors and relevant beliefs, stakeholders, and industry influence.***

***About the interviewee***

1. Could you please tell us a little about your role?
2. What are the main mandate and activities of [your agency]?
  - How would you describe, based on your own opinion, the current priorities?
3. From your perspective, what do you think are the issues or problems facing Fiji, that public policy needs to address?

***In this first section, we would like to ask your opinions regarding current food policy making in Fiji.***

***(Perceptions and understanding of health and nutrition)***

4. From your perspective, what do you think are the major health problems in Fiji?
  - For men? For women?
5. From your perspective, what do you think are the major nutrition problems in Fiji?
  - For men? For women?
6. We are interested in promoting healthy diets to improve health … What do you think are main reasons why people don’t always eat healthy diets (e.g., why do people drink so much sugary drink? Eat salty foods?). [Note: keep this fairly short/minimal – just aiming for main drivers]
  - Prompts: education, economic, culture, religion, geography, food environment, gender
7. Do you think that the government should be actively involved in promoting healthy diets?
  - What do you think is the most effective thing that government can do to improve diets for health in Fiji?
  - Do you think that this will require policy action across a range of sectors?
    i. Which sectors would be most important?
  - Is there a mechanism to coordinate action on nutrition across sectors?
8. From your observation, what do you think are current government objectives with respect to policies and laws on food, agriculture and food system? (Under nutrition? Food security? Obesity? Jobs? Rural development?)
  - Who has responsibility for operationalizing the different objectives, within government? [prompt: sectors and jurisdictions, including subnational]
    i. [e.g., you mentioned rural development: who has responsibility for operationalizing this?]
  - Do you think that nutrition is a priority for government policy making? Why or why not? Does this differ across sectors?
  - In your opinion, how would nutrition compare to other government priorities, like economic growth (more or less important?) or the environment etc. – can you think of other priorities that are probably more important?
  - Do you think food policies that address health issues and diet need to / should consider differences between men and women (for example X and Z)?
  - We have identified a range of policy documents relevant to the food system in Fiji – could you please review these and let us know if you are aware of any we are missing?
    i. Do you think that these policies about food take into account any differences between men and women?
    ii. Additional prompts: e.g., differences between men and women with respect to food provision roles and responsibilities?

***Now we would like to ask about influences on policy for food, agriculture and food systems:***

9. From your observation, how does the government obtain input regarding food policy from citizens, civil society and the private sector?
  - E.g., Formal community consultations, online consultations, stakeholder meetings?
  - Are there different approaches to gaining input for different types of stakeholders?
  - Have you observed informal approaches for input? (E.g., informal meetings)
10. Who are the main actors (people or organizations) with interests in food policy making in Fiji?

- Prompts: Government sectors, Civil Society, Academics, Industry, Development partners
- Use the framework from the stakeholder analysis spreadsheet to ask specifically about different types of interests
- In your opinion, which actors would you say exercise power to influence the policy process?
  - Prompts: Government sectors, Civil Society, Academics, Industry, Development partners
- In what ways have you seen this influence occur?
  - Prompts asking specifically about the different groups in 10(c):
    i. Information and messaging (e.g., lobbying, framing the debate)
    ii. Financial incentives (e.g., funding political parties/major events)
    iii. Constituency building (e.g., establishing relationships with key leaders/organizations/media/community)
    iv. Legal strategies (e.g., legal action - or threat of – regarding public policies)
    v. Policy substitution (e.g., self-regulation or reformulation but on the least consumed products)
    vi. Questioning the basis of policy or developing different sub-groups with different interests

11. I would now like to ask specifically about the food industry. In your capacity as [CEO, Secretary, etc] of [Organization, Ministry, Agency, Development Partner]:
  - Do you or have you interacted or acted with the food industry regarding food policy? If yes, can you describe the situation(s)?
    i. If needed prompt using the strategies in 10(d)
  - If ‘yes’ to the previous question, and from a professional perspective, in what ways do you think the interaction/action(s) might have influenced or impacted policies to improve dietary behaviour? Why?
  - Do you think there should be a formal approach/strategy to recognise and prevent this or these type(s) of interaction(s)/action(s)?
12. The World Health Organization has recommended a range of policy tools, and we wanted to ask specifically about opportunities for strengthening policy related to these: i.e. What opportunities do you see for strengthening the use of …
  i. fiscal policy tools such as taxes and subsidies to promote healthy diets
  ii. Restrictions on unhealthy food marketing to children
  iii. interpretive nutrition labelling on packaged food
  iv. school-based food policies
  v. education and awareness campaigns
  vi. agriculture policies to promote healthy food production
  vii. etc.
13. What are the challenges or barriers to effective implementation of these diet-related policies?

Do you see there being any barriers or enablers in incorporating a stronger gender focus in policies

## Appendix 2

Table 5

**Table 5:** Codes derived from stakeholder interviews

Policy theory		Code	Description
Kingdon’s agenda setting theory	Shiffman’s priority setting theory		
**Problem stream (context)**	**Ideas and frames -**	Current health problems	Respondent comments on the nature of the ‘health problem’ in Fiji, e.g., explanations of prevalence, what the major diseases are etc. This may include gender-specific issues or disease risks.
			Respondent comments on the nature of COVID responses towards NCDS and food and nutrition security
		Current economic problems	Respondent comments on the nature of economic problems and concerns in Fiji, e.g., explanations of challenges or problems faced by the population that are of an economic nature.
			Respondent comments on the nature of COVID and its impact on Fiji’s economy.
		Current other problems (e.g., Inequality, gender issues, equity, natural disasters, climate change etc.)	Respondent comments defining other problems and concerns in Fiji that may impact policy effectiveness.
			Respondent comments on the nature of COVID and the concernsrelating to other issues beyond health and economic development (e.g., Employment, agriculture, gender issues, etc).
		Frames, beliefs, and ideas about the problem (or framing for the challenges and barriers to implementation of NCD policy interventions)	E.g., Economic concerns, awareness, imported foods, cross-sector collaboration, industry tactics and/or corporate political activity, gender equality, etc. This is related to policy process issues in relation to implementation (not policy problems).
	**Issue characteristics features** of the problem: clear measures that show the severity of the problem and which can be used to monitor progress, the size of the health burden and the extent to which the proposed intervention is explained, understood, effective, simple, and inexpensive to implement	Beliefs for root causes of unhealthy dietary behaviours	Respondent comments for perceptions, beliefs and understanding of the root causes of unhealthy diets in Fiji (e.g., apathy, expense, education, imported foods, culture, religion, industry tactics and corporate political activity, gender inequality, conflicting priorities, etc.).
	**Actor power -** This describes policy community coherence, leadership, guiding institutions and civil society mobilisation. It describes the strength of the individuals and networks concerned with the issue, effectiveness of organizations or coordinating mechanisms and the degree of grass-roots support policy actions.	Actor power	Comments on which actors hold power considering different types - formal authority (e.g., govt), informal meetings (potentially relating to industry and corporate political activity) resource-based power (e.g., donors, industry), ideological power (e.g., international organizations, industry) and also how they exercise this power.
		Actor influence	Participants comment on which actors are influential in the development and implementation of NCD policies in Fiji and how they exert their influence (e.g., government ministries, industry, NGOs, FBOs, CSO, etc). In the specific case of industry, it may relate to any of the following: i. Information and messaging e.g.,. lobbying, framing the debate) ii. Financial incentives (e.g., funding political parties/major events) iii. Constituency building (e.g., establishing relationships with key leaders/organizations/media/community) iv. Legal strategies (e.g., legal action - or threat of – regarding public policies) v. Policy substitution (e.g., self-regulation or reformulation but on the least consumed products) vi. Questioning the basis of policy or developing different sub-groups with different interests
		Actor interests	Participants comment on what they perceive to be main interests of the various stakeholders involved in the development and implementation of NCD policies in Fiji.
**Policy stream**		Current health policy priorities	Current health priorities in Fiji and also comments related to the political priority given to NCDs and nutrition in Fiji.
		Current public policy priorities	Current policy priorities in Fiji.
		Current other policy priorities	Current policy priorities competing for attention and resources.
		Current policy commitments	Current policy commitments that may impact attention and resources for health and nutrition policy.
		Policy responsibilities	Respondent comments on why NCDs should be addressed by government and also, which sectors are responsible.
		Perceived political commitment to NCDs	Respondent comments on, from their perspective, the level of political commitment to NCDs relative to other policy responsibilities (e.g., climate change or economic development).
		Perceptions about strengthening the policy or solution (opportunities for strengthened policy implementation)	e.g., focussing on gender issues, youth; awareness through health promotion; education; community involvement; targeting imported food and beverages.
		Use of each intervention/policy: Diet-related taxes	Respondent comments on all the commentary on awareness, effectiveness of existing policies and challenges specific to diet-related polices.
		Restrictions on unhealthy food marketing to children	Respondent comments on all the commentary on awareness, effectiveness of existing policies and challenges specific to restrictions on unhealthy food marketing to children.
		Interpretive nutrition labelling on packaged food	Respondent comments on all the commentary on awareness, effectiveness of existing policies and challenges specific to interpretive nutrition labelling on packaged food.
		Restrictions on school-based food policies	Respondent comments on all the commentary on awareness, effectiveness of existing policies and challenges specific to restrictions on school-based food policies.
		Education and awareness campaigns	Respondent comments on all the commentary on awareness, effectiveness of existing policies and challenges specific to education and awareness campaigns.
		Agriculture policies to promote healthy food production	Respondent comments on all the commentary on awareness, effectiveness of existing policies and challenges specific to agriculture policies to promote healthy food production.
**Political stream**	**Shiffman’s (political) Context** - The environments in which actors operate. **Policy windows:** political moments when conditions align favourably for an issue, presenting opportunities for advocates to influence decision makers. **Governance structure:** the degree to which norms and institutions operating in a sector provide a platform for effective collective action.	Institutional structures	Participants comment on the coherence and coordination of multi-sector actions for the implementation and enforcement of NCD interventions in Fiji.
		Political context	Respondent comments or reflects on significant political events, paradigms or persuasions that are relevant to or affecting nutrition policy.
**Policy entrepreneurs**	**Policy Champions** or advocates taking the policy environment forward in favourable moments such as policy windows	Policy champions	Participants identify specific players they perceive driving nutrition policy in Fiji forward.
	**Resources**	Use of resources	Perceived awareness.
			Perceptions and beliefs on use of donor resources.

## Appendix 3

Table 6

**Table 6:** Active NCD-relevant policies in Fiji

	Policy (years active)	Summary of policy objectives relevant to NCDs	Key policy relevant actions for diet-related NCD prevention
Economy	National Development Plan (2017+)	The plan indicates Fiji will be placing more emphasis on preventative health care by promoting physical activity and other lifestyle changes to reduce NCDs. The plan also includes a strategic action to ‘*establish a framework for a multisectoral approach to address agriculture and NCDs in Fiji*.’ Strategies include more food laws and regulations, a school nutrition policy, health promotion and improved rehabilitation services. **Priority**: Promote population health and reduce premature morbidity and mortality due to NCDs as part of a whole-of-society approach to wellness and well-being. **Strategy: **Expand investment approaches to address NCDs, including nutrition and mental health, within and beyond the health sector.	**Examples of outcomes expected/target for 2019/2020**: • Food Law and additional food regulations and school nutrition policy. • Health promotion for reduced premature mortality due to NCDs. • Rehabilitation services for NCD-related disability and injuries (including through diabetes hubs). • Fiji plan of action for nutrition for exclusive breastfeeding; a 10-year costed survey plan for STEPS, National Nutrition Survey, oral health, prepared and updated annually. **Key indicator**: • Premature mortality due to NCDs to reduce from 68.2% (2015) to 49.7% (2021).
	Fiscal Policy (Budget address/tax schedule) (2019)	This policy provides an overview of Fiji’s current macroeconomic and fiscal position and projections for the next three years (August 2019–July 2022). It also outlines the overall fiscal strategy and direction for the medium term, which is geared towards achieving inclusive economic growth and fiscal sustainability. The tax schedule notes the removal of import duties of fruit and vegetables is to ensure affordability of healthy foods and to promote combating NCDs.	• The 2012 tax schedule (amended 2016) indicated the following items have excise duties (in addition to various fiscal duties): ice cream, powders and preparations for making beverages other than those with the basis of milk, sugar syrups, flavoured waters containing added sugar or sweetening agents (15%); biscuits and sweet biscuits, wafers, toasted bread/crackers (15%); chocolate and chocolate-coated products and cocoa-containing products - and sugar confectionery (15%). • In 2018/2019, the import duty was reduced from 5 to 0% on apples, carrots, grapes, oranges, pears, mixed Vegetables, celery, capsicum, mushrooms, kiwi fruit, asparagus, strawberries, leeks, spinach, apricots, peaches, plum, grapefruit, raspberries, cranberries, pomegranate, cauliflower, broccoli and Brussel sprouts. In 2019/2020 a higher specific rate of duty of 32% or $2 per litre was be applied to imported SSBs. • In 2019/2020, an import excise tax on imported chicken was increased from 0 to 10%.
Health and Medical Services	National Strategic Health Plan (2020–2025)	This policy provides the overall strategic direction for Fiji’s health sector. **Specific objectives** relating to diet-related NCDs are to reduce lifestyle risk factors among the population; the early detection, risk assessment, behaviour change counselling, clinical management, and rehabilitation for targeted NCDs.	**Key indicators**: • No key indicators for the current Health Plan. The previous Plan had outlined the following key indicators: • Reduce premature mortality due to NCDs from 40.7% (2014) to 37% (2020). • Reduce population prevalence of diabetes from 31% (2014) to < 22% (2020). • Reduce prevalence of overweight/obesity in primary school children from 15.7% (2014) to < 10% (2020).
	Non-communicable Diseases Strategic Plan (2015–2019)	Reduction or no increases in all NCDs and their risk factors including mental health, violence, alcohol, physical activity, tobacco. Increased resource allocation as appropriate	**Key Indicators** for diet-related NCD indicators: • Reduced intake of salt per person aged 18+ years by 20% by 2019. • Increased daily average serves of fruit and vegetables among adolescents and adults by 10% by 2019. • No increase in obesity prevalence in adults or adolescents. • No increase in diabetes prevalence in adults.
	DRAFT Fiji Policy on Food and Nutrition Security (2018–2022) (At the time of publication, this policy was not endorsed)	Extensive list of policy objectives encompassing multi-sector leadership, ownership and coordination of national food security actions for sustainable food and nutrition security enhanced maternal and child health, social protection programmes, and supporting healthier school environments, better food standards and nutrition-sensitive food chains	**Key actions** for diet-related NCDs: • Incorporate the Food and Health Guidelines for Fiji into all nutrition and health programs to promote healthy eating and dietary practices for all Fijians. • Create safe and supportive environments for nutrition at all ages. • Promote healthy weight and reduce underweight, overweight and obesity among the population. • Strengthen coordination to implement best practices on fat, sugar, salt reduction strategies in consultation with partners’ e.g., health star rating with food industries, for adoption and submission to Cabinet. • Strengthen implementation and monitoring of community nutrition advocacy campaigns to promote fat, sugar and salt reduction strategies on meals at home and those purchased outside the home, to promote consumption of recommended number of daily serves of fruit and vegetables, healthy foods and increased physical activity, to reduce SSB consumption. • Support the development of healthy recipe books for easily grown, accessible and low-cost local root crops, fish and other local foods to help create interest that would result in increased consumption of these foods by households. • Strengthen multi-sectoral collaboration and advocacy to promote healthy diets and lifestyles within consumer groups, faith-based organisations, non-governmental and civil society organisations. • Strengthen multi-sectoral support, engagement and collaboration on the inclusion of healthy diet and lifestyle information into in-service training programmes to create more enabling institutional environments for improved nutrition outcomes.
	Wellness Policy (2015 )	The Wellness Policy reframes the findings of the NCD STEPS Surveys (in adults) and indicates that although the proportion of the healthy population is decreasing, there is still significant opportunity to encourage healthy lifestyles. The policy is based on a multisectoral, holistic approach to the concept of ‘wellness’ of the entire Fijian population. Its primary objective is to lay out the development of the National Wellness Strategic Plan (2015–2019).	No key indicators noted for diet-related NCDs.
Trade	Fijian Trade Policy Framework	In addition to global concerns over health issues surrounding tobacco products, this policy raises concerns over trade in products that are seen as potentially increasing susceptibility to NCDs.	• No key indicators noted for diet-related NCDs.
Agriculture	Fiji 2020 Agriculture Sector Policy Agenda (2014)	This policy is aimed at exporting agricultural products to international markets although currently, Fiji’s primary market is domestic. The objective of the strategic plan is that through improved infrastructure and processes, high quality goods can be exported overseas, and Fiji will become an internationally competitive central hub for exported agricultural produce.	• No key indicators noted for diet-related NCDs.
Women and Youth	Fiji National Gender Policy (date unidentified)	Generic health access to services diseases causing morbidity and mortality, recognising the need for holistic approaches to health and acknowledging the physical and psychological differences between men and women.	• No key indicators noted for diet-related NCDs.
Education	Fiji School Health Policy (2016 – to be reviewed every two years)	This policy provides generic ‘health and wellness’ activities to be supported and implemented in all schools through inclusion in the school curriculum by Ministry of Education, Heritage and Arts (MoEHA). Also, it implies an integrated approach to School Health Programs (SHPs) within the Ministry of Health & Medical Services’ (MoHMS) public health programs. Finally, it aims for strengthened multi-sectoral collaboration and coordination of wellness activities targeting the health of children in school and coordination of wellness activities	• No key indicators noted for diet-related NCDs.
	Policy on Food and School Canteens (2017 +)	This policy gives clear procedures and expectations by the Ministry of Education, Heritage and Arts (MoEHA) for canteens and where operators or School Heads and teachers are to engage collaboratively towards the provision of healthy food and beverages in the school canteen and to promote health food environments in the school. Supports the principles taught in various year levels which aim to promote healthy eating practices, healthy living, well-being and the safety of all students in school.	• No key indicators noted for diet-related NCDs.

## Abbreviations

FBG: Fiji Beverage Group
FCTC: Framework Convention on Tobacco Control
FDA: Food and Drug Administration
GBD: Global Burden of Disease
NCDs: Non-communicable diseases
NDP: National Development Plan
NGOs: Non-government Organizations
NHMRC: National Health and Medical Research Council
SSBs: Sugar-sweetened beverages
SDGs: Sustainable Development Goals
WHO: World Health Organization
WPRO: WHO Western Pacific Region
