# Supplementary figures and images for: Strengthening implementation of diet-related non-communicable disease prevention strategies in Fiji: a qualitative policy landscape analysis
Source: Global Health. 2022 Sep 1;18:79. doi: 10.1186/s12992-022-00859-9 (PMC9434519; doi:10.1186/s12992-022-00859-9)

Additional file 1. WHO policy recommendations for NCD prevention
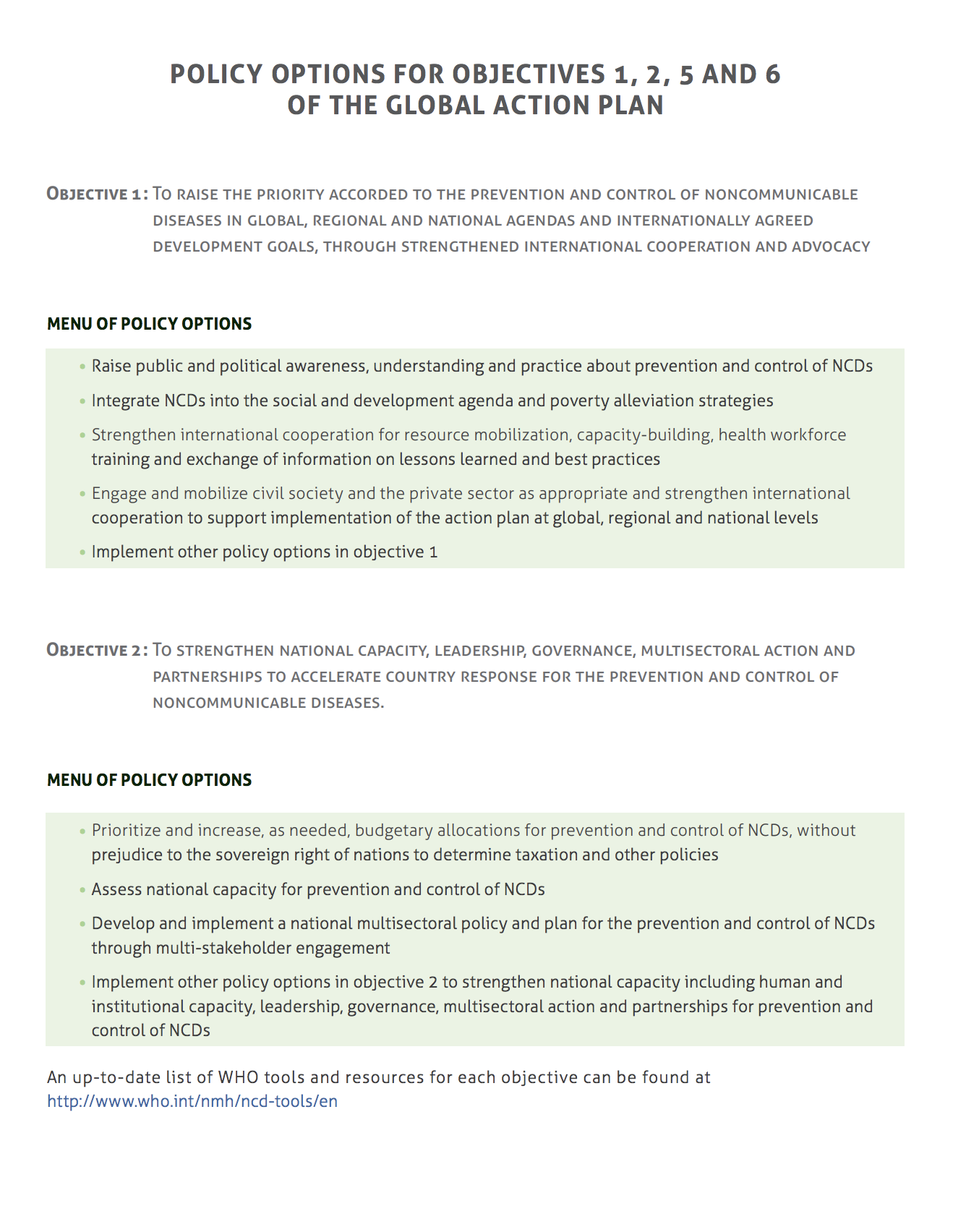


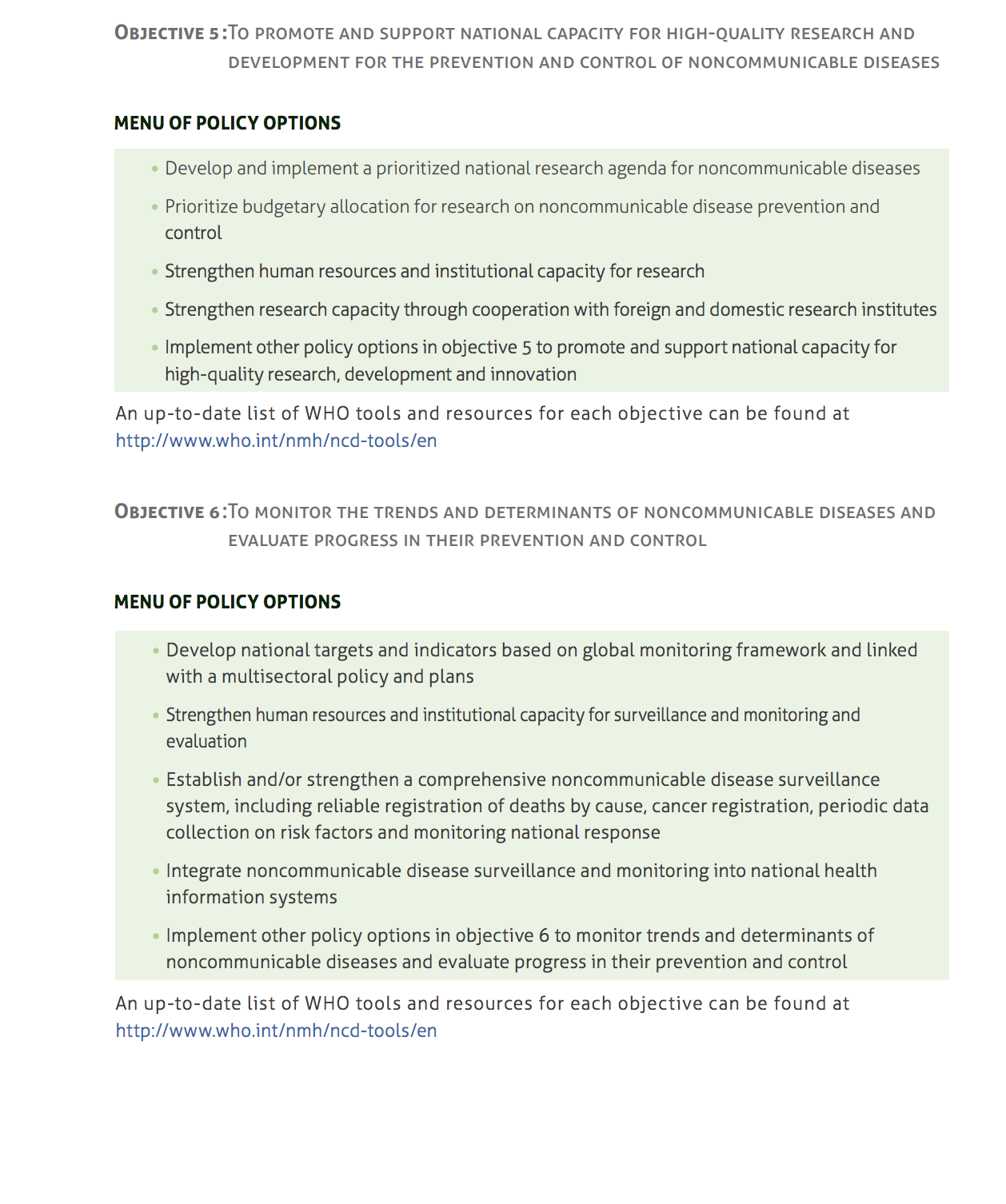


Source: WHO (2017) [[3](#_ENREF_3)]

Supplement: Supplementary file 1 — Additional file 1. WHO policy recommendations for NCD prevention. Source: WHO (2017) [3]. [file 12992_2022_859_MOESM1_ESM.docx]
